# Wiki-Quantities and Wiki-Measurements: Datasets of quantities and their measurement context from Wikipedia

**DOI:** 10.1038/s41597-025-05499-3

**Published:** 2025-07-22

**Authors:** Jan Göpfert, Patrick Kuckertz, Jann M. Weinand, Detlef Stolten

**Affiliations:** 1 Forschungszentrum Jülich GmbH, Institute of Climate and Energy Systems, Jülich Systems Analysis, 52425 Jülich, Germany; 2 RWTH Aachen University, Chair for Fuel Cells, Faculty of Mechanical Engineering, 52062 Aachen, Germany

**Keywords:** Research data, Computational science, Scientific data

## Abstract

To cope with the large number of publications, more and more researchers are automatically extracting data of interest using natural language processing methods based on supervised learning. Much data, especially in the natural and engineering sciences, is quantitative, but there is a lack of datasets for identifying quantities and their context in text. To address this issue, we present two large datasets based on Wikipedia and Wikidata: *Wiki-Quantities* is a dataset consisting of over 1.2 million annotated quantities in the English-language Wikipedia. *Wiki-Measurements* is a dataset of 38 738 annotated quantities in the English-language Wikipedia along with their respective measured entity, property, and optional qualifiers. Manual validation of 100 samples each of Wiki-Quantities and Wiki-Measurements found 100% and 84-94% correct, respectively. The datasets can be used in pipeline approaches to measurement extraction, where quantities are first identified and then their measurement context. To allow reproduction of this work using newer or different versions of Wikipedia and Wikidata, we publish the code used to create the datasets along with the data.

## Background & Summary

As the volume of publications continues to grow, scientists are increasingly challenged to keep track of their research field. Information extraction is used to transform aspects of publications into structured information to enable large-scale literature analyses^1,2^. Much of the data, especially in the natural and engineering sciences, is quantitative, and therefore measurement extraction is of great importance. While information extraction traditionally focuses on identifying mentions of named entities and the relationships between them, measurement extraction specifically deals with the identification of quantities and their measurement context, such as their measured properties and entities^3^. In a previous review, we show that measurement extraction has received little attention in the history of information extraction, which is reflected in a lack of large, high-quality datasets for extracting quantities and their measurement context^3^. As measurement extraction is typically approached with supervised learning, the lack of annotated data limits the performance of measurement extraction systems. The task of measurement extraction is typically approached in a pipeline manner, where 1) quantities are identified before 2) their individual measurement context is extracted^3^. To support the development and evaluation of measurement extraction systems, we present two datasets^4^ that correspond to the two tasks:

- **Wiki-Quantities**, a dataset for identifying quantities, and
- **Wiki-Measurements**, a dataset for extracting measurement context for given quantities.

**Fig. 1:**
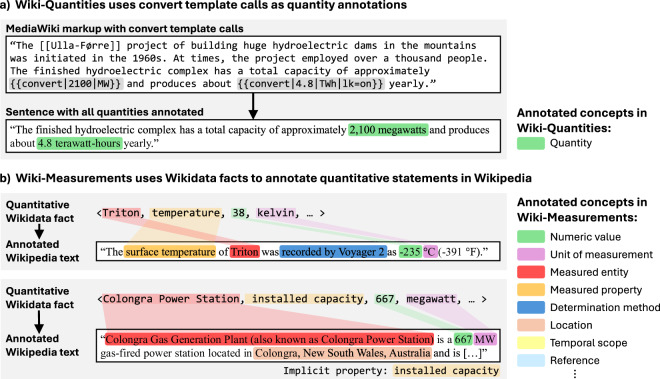
Schematic illustration of the methodology used to create the datasets: (**a**) Wiki-Quantities uses {{convert}} template calls as quantity annotations. (**a**) Wiki-Measurements aligns Wikidata facts with text from their respective Wikipedia page. Both implicit and explicit properties are supported. The example texts are from Wikipedia^55– 57^.

**Fig. 2:**
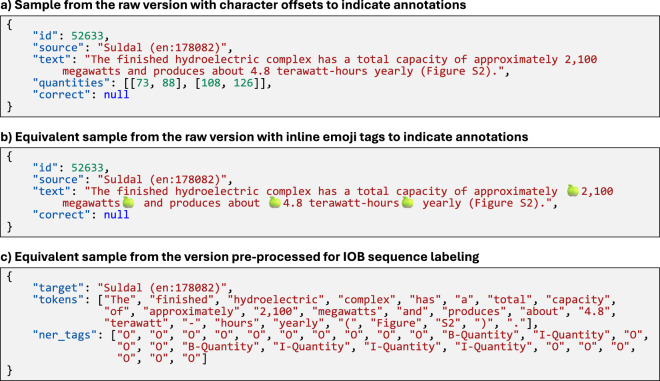
Wiki-Quantities is provided in different formats: (**a**) using character offsets to indicate the start and end of annotations, (**b**) using emoji tags to indicate annotations and facilitate curation, and (**c**) pre-processed for IOB sequence labeling. source indicates the source Wikipedia page of the example text. correct indicates the curation status: if set to true, the quantity annotations were considered correct during manual curation; if set to false, the quantity annotations were considered incorrect; if set to null, no manual curation was performed. The example text is from Wikipedia^55^.

**Fig. 3:**
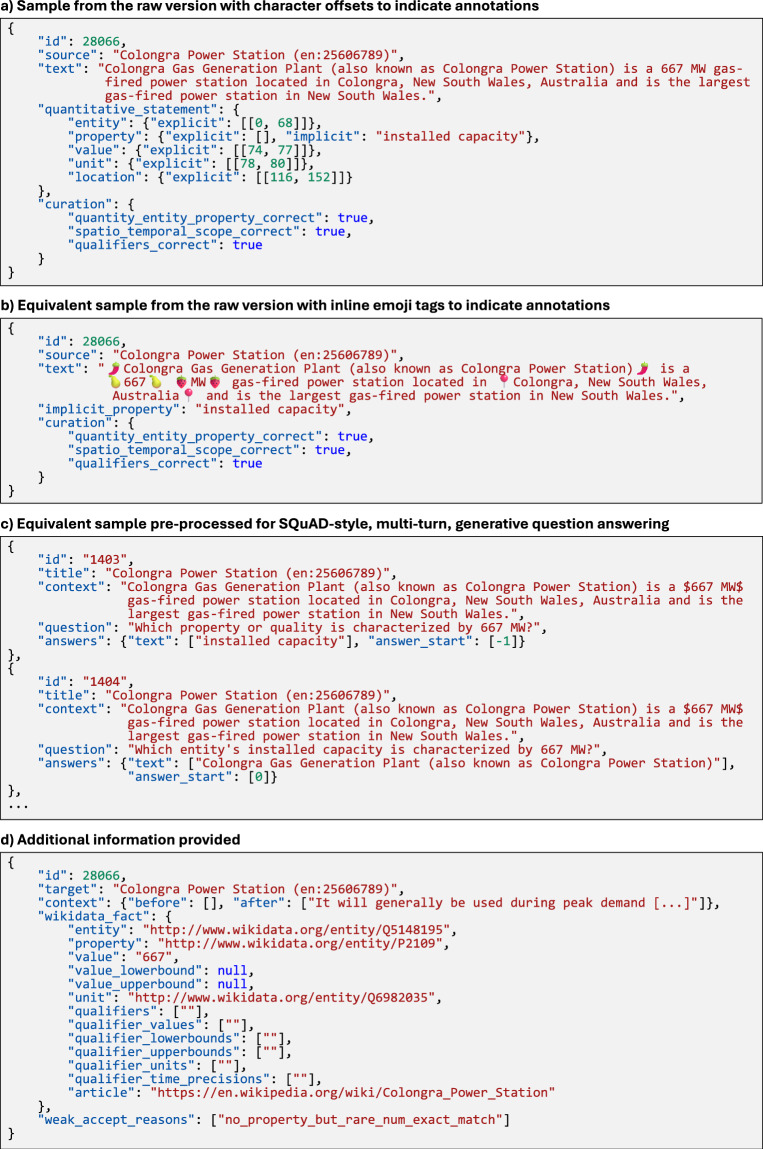
Wiki-Measurements is provided in different formats: (**a**) using character offsets to indicate the start and end of annotations, (**b**) using emoji tags to indicate annotations and facilitate curation, and (**c**) pre-processed for SQuAD-style, multi-turn, generative question answering. Additional information (**d**) provides the Wikidata fact with URIs for its items, surrounding text, and, if not all quality criteria were met when matching the Wikidata fact to the Wikipedia article, the reasons why the example was accepted anyway. The mapping of emojis to annotation types is given in the README of the dataset. source indicates the source Wikipedia page. Unlike the other annotations, the measured property can be given as a string in the implicit field if it is not explicitly given in the text, or to correct its spelling, increase its information value (e.g., ‘surface area’ instead of ‘surface’), or adjust its inflection (e.g., ‘length’ instead of ‘long’). Entity, property, value, and unit annotations are mandatory, but may be empty if no corresponding span is found in the text. The annotation of qualifiers and quantity modifiers is optional. If quantity_entity_property_correct is set to true, the quantity, entity, and property annotations were considered correct during manual curation. If spatio_temporal_scope_correct is true, the temporal and spatial scope were considered correct. If qualifiers_correct is true, all qualifiers were considered correct. If any of the three fields is false or null, the corresponding annotations were considered incorrect or no manual curation was performed, respectively. For the pre-processed version, a separate example with the corresponding formatting, question, and answer is provided for each annotation type. The pre-processed data implies that for a given quantity, first the measured property is asked for, then the measured entity, and finally the qualifiers. Previous answers are highlighted in the context using dollar signs for the quantity, double asterisks for the measured property, and double brackets for the measured entity. For the measured property, the implicit property is taken as the answer if it is not empty. In this case, the answer_start is set to -1. The example text is from Wikipedia^57^.

In Wiki-Quantities, each example is a sentence in which all quantity spans are annotated, such as the example in Fig. 1a. In contrast, Wiki-Measurements consists of sentences in which the measured entity (e.g., Eiffel Tower) and property (e.g., height) are annotated for a single quantity (e.g., 330 m). The measured property may be given implicitly or explicitly (see Fig. 1b). Qualifiers such as temporal or spatial scopes or measurement methods and quantity modifiers such as ‘approximately’ or ‘at least’ are considered optional. Therefore, qualifier and quantity modifier annotations are only sparsely included. In Wiki-Measurements, quantity annotations are divided into value and unit annotations. See Figs. 2, 3 for sample data from both datasets. The datasets are based on text from Wikipedia pages, annotated based on information in their MediaWiki markup for Wiki-Quantities, and based on the alignment of facts from their corresponding Wikidata entry for Wiki-Measurements (see Fig. 1). Wikidata^5^ is an openly licensed, collaboratively developed knowledge graph and, as Wikipedia, it is part of the Wikimedia Foundation. At the time of writing, Wikidata contains information on more than 117 million items^6^, including many quantitative statements such as  < Airbus A380, wingspan, 79.8, metre > ^7^.

In the following, we give an overview of related datasets, all of which either have a different scope or are considerably smaller.

### Datasets of annotated numerals and quantities

Numerical concepts such as percentages, monetary expressions and quantities have long been part of tag sets in named entity recognition (NER) datasets^8–10^. Today, several datasets are specifically targeted at numerical information. Many of these, such as *Numeracy-600K*^11^, *NumerSense*^12^, and others^13,14^, contain annotations of numerals without units of measurement. However, many numerals are not part of a quantity (e.g., 15 in ‘iPhone 15’). The applicability of these datasets for measurement extraction is further limited by considering only numerals that are written out (i.e. ‘ten’ instead of ‘10’)^12^, omitting numerals outside a range^14^, not including mathematical symbols and therefore ignoring signs^14^, or splitting fractions into multiple numerals^13^.

In contrast, *Grobid-quantities*^15^ distinguishes between quantities and other numerals. In addition, quantities are classified into different types (e.g., list, range, least, most, etc.). Both the numeric value and the unit of a quantity are annotated. The numeric value is further subdivided into exponent, power, base, time expressions, and alphabetic numbers. The unit is further divided into prefix, base, and power. However, at the time of writing, the dataset consists of only 35 labelled documents^16^. Similarly, *MeasEval*^17^ distinguishes between quantities and other numerals, annotates both the value and the unit, and classifies a quantity into different types (e.g., range, list, approx., mean, etc.), but only consists of 428 annotated paragraphs. *CQE*^18^ provides a test set that lists all normalized quantities per sentence, but does not indicate their position. Other datasets distinguish between numeric values and units, but only annotate certain quantities^19^.

The aforementioned datasets that rely on human labeling are relatively small, and those that are based on rule-based approaches do not distinguish between numerals as part of a quantity, nominal numbers (e.g., ZIP codes), and ordinals. In contrast, the approach presented in *Wiki-Convert*^20^ allows for both a large dataset and the distinction of quantities from other numeric expressions. In Wiki-Convert, annotations are drawn from {{convert}} template calls in the English Wikipedia, so numbers are more likely used as quantities. Wiki-Convert is published in a version where the {{convert}} template calls are transformed into readable text but only the number is annotated, not the unit, and sentences with multiple number annotations are dropped as well as those where the {{convert}} template involves a unit outside the 30 most common units^20^. The dataset is published in another version without these limitations, but without converting the template calls to readable text. In both versions, quantities are sparsely annotated, making them unsuitable for sequence labeling approaches to identifying quantities in text. Wiki-Quantities^4^ is a re-creation of Wiki-Convert that removes these limitations, is readily usable for sequence labeling, and is published with the code to enable its recreation. The difference in the number of examples (832k for Wiki-Quantities vs. 925k/1.34M for Wiki-Convert) results from the different filter rules applied for the distinct use cases. In particular, Wiki-Quantities only retains examples for which all quantities are annotated.

### Datasets of annotated quantities and their context

Going beyond mere numerals, several datasets such as *NumER*^21^, *FinNum 1.0*^22^, *FiNER-139*^23^, *NumClaim*^24^, and *ECNum*^25^ additionally state the numeral type (e.g., age, population, or money), yet without including units in the annotations. Other datasets focus on specific entities and quantities: *SuperMat Corpus*^26^ focuses on mentions of superconducting materials and their critical temperature; the *SOFC-Exp Corpus*^27^ focuses on materials, quantities, and devices describing experiments related to solid oxide fuel cells; and the *Materials Science Procedural Text Corpus*^28^ focuses on materials synthesis procedures. Other datasets, such as *BioNumQA*^29^, *NQuAD*^30^, *FinQA*^31^, *DROP*^32^, or datasets of math word problems require models to perform numerical reasoning and mathematical operations, but do not focus on detailed annotation of quantities and their context.

*MeasEval*^17^, already mentioned as a dataset of annotated quantities, additionally provides annotations for their measured entities and properties as well as qualifiers. Qualifiers describe further context that is relevant to the particular quantification. However, the dataset consists of only 428 labeled paragraphs, which limits the performance of machine learning models trained on it^33^. *Chaudron*^34^ is a dataset of measurements extracted from Wikipedia infoboxes. Methodologically related to our approach, Wikipedia infoboxes have been used to heuristically generate training data from the articles in which they are embedded in^35^. Similarly, *LUCHS*^35^ is a system for extracting triples from text that is distantly supervised by aligning facts from Wikipedia infoboxes with the corresponding article. Most similar to our work, Chia *et al*.^36^ and Chen *et al*.^37^ align Wikidata^5^ facts with Wikipedia articles to create datasets for general hyper-relational fact extraction and temporal fact extraction, respectively. Although they do not focus on quantitative facts, a subset of the datasets describes quantitative facts. However, these are limited to hyper-relational and temporal facts, respectively, and as the focus is not on quantitative facts, unlike Wiki-Measurements^4^, the matching of Wikidata facts with Wikipedia articles is done without accounting for the diverse representation of quantities due to different levels of rounding and unit conversion, among others.

## Methods

Many systems for measurement extraction first identify quantities before extracting their measurement context^3^. This pipeline approach not only results in powerful systems but also facilitates the creation of datasets. Whereas a dataset for quantity span identification must provide annotations for all quantities that occur in a text, a dataset for measurement context extraction may provide sparse annotations for quantities, but for given quantities must provide complete annotations of their measurement context. In the following subsections, we describe the largely automated creation of large datasets for quantity and measurement context extraction^4^, which is made feasible due to their different requirements. To improve the reproducibility of this work, all code is published open-source and accompanied by a Snakemake^38^ workflow that formalizes all the steps involved in generating the raw datasets. In addition, the datasets have been deduplicated, balanced, and partially manually curated.

### Wiki-Quantities

Wikipedia authors use templates to reuse frequent content or to automate certain functions when editing Wikipedia articles. Similar to function calls in programming, templates are invoked by specifying their name and arguments in double curly braces directly within the MediaWiki markup, which can be thought of as the source code of a Wikipedia article. When rendering the HTML representation of a Wikipedia article, MediaWiki replaces template calls with their returned values. One such template is the {{convert}} template which is used to automatically convert quantities from one unit to another. For example, the string convert|11|m/s|km/h|abbr=on^39^ will be rendered as “11 m/s (40 km/h)”^40^. More complex calls are also supported. For example, convert|60-62.5|m|ft+royal cubit|…^41^ is displayed as “60-62.5 m (197-205 ft; 115-119 cu)”^42^. To create the quantity dataset, we take {{convert}} template calls in the MediaWiki markup as quantity annotations, as previously described in Thawani *et al*.^20^.

#### Parsing MediaWiki markup and {{convert}} template expansion

For each article in the English Wikipedia and Simple English Wikipedia, we parse the MediaWiki markup into readable text. We use database dumps from Q3 2022. The Simple English Wikipedia is written with simplified vocabulary and grammar and is used additionally to increase the amount of annotated data. Potential duplicate data records are later removed. When parsing MediaWiki markup, we remove infoboxes, sidebars, references, and comments, and expand all {{convert}} templates, that is, replace the template calls with the results returned by the corresponding {{convert}} module. For this, we isolated the {{convert}} module written in the Lua language from MediaWiki, in which it is interwoven, and invoke it directly from within Python. The returned strings are taken as the quantity annotations. Any template calls that reference Wikidata are ignored. We customized the {{convert}} module to return three versions of a quantity string: the quantity in input units, the quantity in output units, and both combined into one string. We randomly select one of these versions, giving the input and output quantities the same probability of 47.5%. The combined string is only given a probability of 5% because it occurs less frequently in other corpora.

#### Post-processing

We split the text into sentences using spaCy^43^ and keep only those that include at least one quantity annotation. To ensure these do not include further quantities that were not specified using the {{convert}} template and therefore not annotated, we filter out sentences where digits, number words, and so forth appear outside the quantity annotations. However, based on the NER annotations provided by spaCy, we allow products, dates, organizations, and so forth to contain numerals. We drop sentences containing “per” outside of quantity annotations to filter out incomplete quantities such as “[2 million barrels]_Quantity_ per day”, as well as sentences shorter than 10 characters.

To provide a training incentive not to confuse citation and reference spans (e.g., ‘[1]’, ‘^2-3^’, ‘Fig. IV’ or ‘Table A1’) with quantities, we add citation and reference spans to the end of sentences. We sample a diverse and realistic set of citation and reference spans from the S2ORC corpus^44^—a large dataset of scientific articles that has been parsed into a machine-readable format. We add phrases like “cf.” or “as shown in” to some of them and enclose them in parentheses () or brackets [], where the probability of using parentheses or brackets depends on the type of citation or reference. For example, citations such as “Einstein *et al*., 2024” or references to figures and tables are less likely to be enclosed in brackets than numerical references. We place all figure and table references (e.g., “Fig. 1”) before the full stop and bibliographic references (e.g., “[1]”) after the full stop with a 5% probability.

To ensure that the dataset is readily usable for training sequence labeling models, many of which have a context window limited to 512 word-piece tokens, we split examples that exceed this length into multiple examples. To determine the number of tokens, we use the Hugging Face^45^ implementation of the RoBERTa^46^ tokenizer.

We manually curated a subset of the data and corrected the entire dataset for common errors. This included removing empty parentheses ("(;)”), deleting repetitions of units ("3.1 metres metres”), removing ‘-high’, ‘-long’, etc. from the quantity span ("3.1-meter-high”), and expanding the quantity annotation where necessary ("’1 meter’ square”, “’3.1 in’ for every second”, “’38 °C’ ± 1”). The result is a high-quality dataset^4^ of sentences with annotated quantities.

#### Deduplication and balancing

However, the raw dataset is imbalanced with respect to units. Expressions for meter, kilometer, feet, inch, miles, acres, kilogram, °C, km/h, mph, and so forth, are predominant. Accordingly, the dataset is dominated by the description of lengths, heights, distances, weights, and so forth. Additionally, many of the most frequent words either describe properties directly (such as ‘long’, ‘length’, ‘highest’, ‘speed’, or ‘altitudes’) or limit the number of properties to expect by setting a topic (such as ‘river’, ‘lake’, ‘town’, ‘mountain’, or ‘highway’). In addition, the dataset contains duplicates and many similar examples, which we hypothesize are the result of bots and templates being used to create Wikipedia pages for certain frequent but repetitive article classes, such as road networks, rivers, mountains, municipalities, lighthouses, and so on.

We remove examples that exceed a certain similarity threshold in their normalized form, where normalization includes converting all characters to lowercase, replacing all quantity spans with a special character, removing leading and trailing whitespace as well as references at sentence ends, replacing all directions with ‘north’, all years with ‘2023’, all months with ‘January, and common terms referring to settlements (e.g., city or village) with ‘region’. As a measure of similarity, we use the token-based Levenshtein distance^47^. Tokenization is done by splitting on whitespace. If the absolute value of the edit distance is greater than 35% of the number of tokens, the example is considered unique, otherwise it is removed from the dataset. In this way, we removed 37 530 duplicate examples and a further 233 771 examples based on the edit distances, which equates to a 32.6% reduction in the number of examples. 561 393 examples remain. We refer to the original dataset as Wiki-Quantities (raw) and to this scaled-down version as Wiki-Quantities (large). Because Wiki-Quantities is orders of magnitude larger than other quantity span identification datasets, reducing its size helps avoid overfitting to it in scenarios where a model is trained on a mixture of quantity span identification datasets.

To balance the dataset with respect to the units, properties, and topics, we remove examples based on unit and token counts. For this, we compile a list of the most frequent tokens, remove neutral tokens (e.g., quantity modifiers, such as ‘approx.’, and stopwords), and randomly remove examples that contain any of them. The probability increases with the frequency of the token and is given by 1 − *t*_*thr*_/(*t*_*i*_ + *t*_*thr*_), where *t* is the number of occurrences of token i in the dataset and *t*_*thr*_ a parameter to control the degree of filtering. Hereinafter, we limit the number of examples containing a quantity span ending on a specific unit in the dataset to a threshold count of *u*_*thr*_. Both *u*_*thr*_ and *t*_*thr*_ control the degree of filtering. Depending on their value, we get datasets of different sizes. In addition to Wiki-Quantities (raw) and Wiki-Quantities (large), we publish a small and tiny variant corresponding to a (*u*_*thr*_,  *t*_*thr*_) of (1200, 1000) and (300, 1000), respectively (see Table 1). Table 2 compares the occurrence frequencies of units in the Wiki-Quantities variants. As to be expected, Wiki-Quantities (small) and (tiny) are more balanced with respect to units than the other variants.

**Table 1:** Dataset statistics for different variants of Wiki-Quantities.

	Wiki-Quantities (raw)	Wiki-Quantities (large)	Wiki-Quantities (small)	Wiki-Quantities (tiny)
#Quantities	1 227 626	779 943	59 504	26 076
#Examples	832 660	561 393	44 729	20 148
#Curated Examples	289	289	289	289
#Wikipedia Pages	434 860	289 217	35 758	17 006

### Wiki-Measurements

Distant supervision is an approach to deal with data scarcity in relation extraction tasks by labeling relations between entities based on known triples in a knowledge base^48^. The creation of the measurement context dataset^4^ is based on distant supervision, where matches to a knowledge base are treated as (somewhat noisy) ground truth. Our knowledge base consists of quantitative facts from Wikidata^5^. Wikidata publishes RDF dumps that can be queried via its SPARQL endpoint (https://query.wikidata.org/). RDF is a data model standardized by the World Wide Web Consortium, in which information is represented in triples containing a subject, a predicate and an object. We query Wikidata for quantitative statements and align these with associated Wikipedia articles to create text chunks with annotated quantity, property, entity, and optionally qualifiers.

#### Obtaining quantitative statements from Wikidata

First, we query Wikidata for statements whose subject is an item that has an associated article in the English or Simple English Wikipedia, whose predicate is of type ‘quantity’, and whose object is composed of a numeric value and a unit. Counts, that is, quantities without a unit, are also considered, as they have the unit ‘1’ in Wikidata. Additionally, we get the lower and upper bounds of the numeric value, if specified. We also query for qualifiers that constrain quantitative statements, for example, in terms of measurement method, date, or precision. Qualifiers may be qualitative or quantitative. In the latter case, we again retrieve the numeric value, the unit of measurement, and the lower and upper bounds. For temporal qualifiers, we get the time and its precision (such as year or day). When we hit the timeout of Wikidata’s public SPARQL endpoint, we use the results obtained up to that point, as this naturally balances the dataset and we can do without loading the Wikidata dump into a local triplestore. We retrieve 636 different predicates of type ‘quantity’. Limiting the number of results per predicate to 250 000, we retrieve 3 018 117 and 622 295 quantitative statements for the English and Simple English Wikipedia, respectively. The queries are given in Query 1 and 2.

#### Aligning Wikidata facts and their associated Wikipedia articles

Next, we align the quantitative statements obtained from Wikidata with text chunks of their associated Wikipedia articles. Analogous to the creation of Wiki-Quantities, we parse the MediaWiki markup into plain text, perform sentence segmentation, and expand {{convert}} template calls. To be considered a valid example of the dataset, a sentence must contain the numeric value as well as the label or any alternative label of the measured entity, property, and unit. As properties are often given implicitly, a matching property span is considered optional if other evidence provides sufficient confidence that the quantitative statement is correctly aligned with the Wikipedia page, i.e., an entity match is required and the numeric value must be infrequent (that is, not 1-10) and match exactly. In this case, we use the Wikidata statement’s property label as the annotation.

##### Query 1

SPARQL query used to get all quantitative properties in Wikidata.

##### Query 2

SPARQL query used to get up to {limit_threshold} quantitative statements for a property {property_id} in Wikidata.

To match the numeric value, we attempt to identify all numbers using a combination of regular expressions, gazetteers, and rules on “CD” and “like_num” tags provided by spaCy^43^. We filter out ordinals, dates, and numbers preceded by “per”. We perform **approximate matching** of numeric values, that is, if bounds are given, the numeric value must lie within the corresponding interval, otherwise the numeric value is allowed to deviate by a mean absolute percentage error (MAPE) of at most 3% in order to match. Additionally, we perform **unit conversions** to increase recall. The same quantity can be expressed in different units of measurement, hence we convert the quantities of the Wikidata statements into various units when matching them. For performance reasons, we only consider unit conversions that have a conversion factor in the interval (10^-3^; 10^3^). For counts, we take succeeding noun phrases as unit (e.g., ‘seat’ in “a 2,564-seat concert hall”). A number of rules improve the alignment precision: Units may not be preceded by strings such as “of which”. If there are multiple matches for a value in the same sentence, but only one is an exact match that is not a count (that is, without a unit), that one is taken. Otherwise, none is taken because there is not enough evidence to decide which value is correct. If a single unit matches that is not adjacent to the target value, it is not allowed to be adjacent to any other value, nor is the target value allowed to be adjacent to any other unit. If multiple units match, but only one is adjacent to the value, that one is taken. Otherwise, the example is discarded.

The labels of units, measured entities, and properties are matched based on their lemmas. Depending on the concept type, annotation spans are expanded based on dependency parsing, part-of-speech tags, and/or NER annotations. As width, height, depth, and thickness are common properties that are often used interchangeably, we use rules to prevent them from being mistakenly associated. If multiple spans match an entity or property, either the one that is in the same subclause as the other annotations or the one that gives the shortest overall path between annotations based on dependency parsing is chosen.

Additionally, we try to match **qualifiers** that we consider optional. We distinguish the following qualifiers (Wikidata property IDs in parentheses): point in time (P585), start time (P580), end time (P582), location (P276), coordinate location (P625), applies to part (P518), in scope of (P642), criterion used (P1013), determination method (P459), and according to (P3680). All other qualifiers are placed under a generic label ("other qualifiers”). Geographic coordinates and temporal scopes are matched using special rules. We do not match rankings (P1352), which are qualifiers used to rate the quality of conflicting statements within Wikidata. For values less than ten, we discard examples without qualifiers because small integers are common and therefore more likely to be mistakenly matched.

After obtaining all examples for a Wikipedia page, we remove duplicate examples and **resolve contradictions**. Sometimes different facts lead to conflicting annotations for a sentence, or the same training example is created multiple times, because there are multiple similar coexisting statements about a property (e.g., the area of Alabama is stated to be 135 765, 134 000 ± 500 or 131 365 ± 0.5 square kilometres^49^). Comparing a pair of examples for the same sentence at a time, the following filter rules are applied: If two examples are identical, we drop one of them. If both match, but one has additional qualifiers, the one with fewer qualifiers is dropped. If both have qualifiers defined and they contradict each other, both are dropped. If two examples share the same value but differ in their entity, property or unit annotation, or if the entity, property, unit and qualifier annotations match but the values do not, both examples are dropped.

#### Deduplication and balancing

We remove duplicates and refer to the dataset as Wiki-Measurements (large). Because the raw dataset differs from the large dataset by only 72 duplicates, the raw dataset is not published. A variant with near-duplicates removed is referred to as Wiki-Measurements (small). In addition, we publish variants where the quantitative facts are more strictly aligned with the text (large-strict, small-strict). In this case, we do not accept examples where the property cannot be matched but a rare numeric value is exactly matched, where the entity can only be matched using co-reference resolution, where a single annotation is selected based on shortest path, or where subclause membership is used. In addition, the threshold for small rounded values is lowered. Furthermore, we publish variants with preceding and following sentences added around the annotated sentence (small-context, small-context-strict). The published dataset is accompanied by additional information for each example, detailing the Wikidata fact, the weak acceptance reasons if any, and the context before and after the annotated sentence, allowing anyone to create further variants (see Fig. 3d).

For further details, the reader is referred to the published source code. The result is a dataset^4^ of sentences, each annotated with a single quantity and its measurement context.

## Data Records

The datasets^4^ are available in the Zenodo repository (10.5281/zenodo.15462002). The format of the data records is shown and described in Figs. 2, 3 as well as in the README file of the datasets.

We publish different variants of **Wiki-Quantities**: The raw variant, the large variant where duplicates and similar examples are removed, and two further filtered variants, referred to as small and tiny, which are more balanced in terms of units and topics. In addition, we publish the data in a raw format and pre-processed for IOB sequence labeling. For the different variants of Wiki-Quantities, Table 1 lists the number of quantity annotations, examples, and Wikipedia pages they originate from. In total, the raw, large, small and tiny variants contain 832660, 561 393, 44 729, and 20 148 examples, respectively. 289 examples have been manually curated and are included in all variants. The raw dataset contains 1 227 626 quantity annotations in 434 860 different Wikipedia pages. Table 2 lists the 50 most frequent units for the different Wiki-Quantities variants. Note that unlike Wiki-Measurements, Wiki-Quantities does not include separate value and unit annotations. Hence, the units are determined by rule-based parsing of the quantity span. Different expressions for units of length (mi, km, etc.), area (acres, km2, etc.), velocity (km/h, mph, etc.), temperature (^∘^F, ^∘^C), mass (kg, lb, etc.), volume (m3, L, etc.), and power (kw, hp, etc.) predominate. The approach to balancing the dataset is effective in leveling the unit shares. Table 3 compares how many quantity annotations and examples are drawn from the English language and Simple English language Wikipedia, respectively. With only 1.2% of all examples stemming from the Simple English Wikipedia, the standard English variant clearly predominates. In Wiki-Measurements (raw), 4.4% of all examples are drawn from the Simple English Wikipedia.

**Table 2:** Top 50 most frequent units for different Wiki-Quantities variants.

	Wiki-Quantities (raw)	Wiki-Quantities (large)	Wiki-Quantities (small)	Wiki-Quantities (tiny)
1	mi (12.45)	m (9.68)	kW (3.27)	kW (2.19)
2	km (10.94)	km (9.53)	km/h (2.73)	km (1.85)
3	m (7.93)	ft (9.05)	km (2.69)	km/h (1.7)
4	ft (7.65)	mi (8.14)	hp (2.66)	PS (1.65)
5	in (6.67)	in (6.47)	m (2.58)	mi (1.63)
6	kilometres (5.97)	miles (4.81)	^∘^C (2.57)	hp (1.6)
7	miles (3.97)	metres (4.23)	kg (2.52)	m (1.58)
8	metres (3.47)	feet (4.19)	acres (2.49)	mph (1.53)
9	feet (3.32)	kilometres (4.09)	lb (2.48)	kg (1.49)
10	mm (3.27)	mm (3.24)	mph (2.46)	^∘^C (1.47)
11	acres (2.2)	acres (2.93)	^∘^F (2.4)	acres (1.47)
12	km2 (2.07)	cm (2.36)	ft (2.34)	ft (1.46)
13	cm (1.96)	km2 (1.96)	mm (2.32)	^∘^F (1.46)
14	sq mi (1.73)	ha (1.7)	mi (2.28)	g (1.45)
15	km/h (1.62)	^∘^F (1.67)	ha (2.21)	t (1.45)
16	^∘^F (1.46)	^∘^C (1.65)	cm (2.21)	L (1.44)
17	mph (1.45)	km/h (1.47)	m2 (2.2)	lb (1.43)
18	^∘^C (1.44)	kg (1.29)	km2 (2.16)	in (1.38)
19	ha (1.24)	mph (1.29)	in (2.16)	m3 (1.38)
20	kg (1.19)	m2 (1.23)	metres (2.09)	mm (1.36)
21	lb (1.05)	sq mi (1.18)	kilometres (2.07)	pounds (1.33)
22	m2 (0.86)	lb (1.17)	miles (2.06)	kilometres (1.33)
23	knots (0.76)	inches (0.9)	feet (2.05)	kilograms (1.31)
24	inches (0.69)	hectares (0.68)	inches (1.91)	feet (1.31)
25	centimetres (0.66)	centimetres (0.67)	long tons (1.71)	cm (1.31)
26	millimetres (0.66)	millimetres (0.66)	t (1.5)	ha (1.31)
27	long tons (0.64)	square kilometres (0.5)	pounds (1.43)	m2 (1.31)
28	square kilometres (0.59)	kW (0.48)	PS (1.42)	millimetres (1.31)
29	hectares (0.57)	meters (0.44)	hectares (1.18)	miles (1.3)
30	nautical miles (0.54)	sq ft (0.44)	m3 (1.04)	km2 (1.3)
31	meters (0.49)	nautical miles (0.41)	short tons (1.04)	hectares (1.28)
32	square miles (0.44)	pounds (0.4)	g (0.92)	centimetres (1.26)
33	kW (0.44)	hp (0.35)	millimetres (0.89)	metres (1.26)
34	t (0.42)	square miles (0.35)	yards (0.87)	psi (1.17)
35	pounds (0.34)	long tons (0.34)	kilograms (0.83)	yd (1.14)
36	sq ft (0.33)	square feet (0.33)	centimetres (0.83)	meters (1.14)
37	kilometers (0.3)	knots (0.32)	L (0.78)	knots (1.13)
38	hp (0.27)	kilometers (0.32)	N ⋅ m (0.7)	inches (1.12)
39	g (0.24)	yards (0.3)	knots (0.69)	yards (1.1)
40	square feet (0.23)	g (0.3)	miles per hour (0.68)	long tons (1.1)
41	tonnes (0.22)	t (0.29)	tonnes (0.66)	tonnes (1.09)
42	oz (0.21)	mile (0.27)	yd (0.65)	oz (1.01)
43	yards (0.2)	kilograms (0.24)	oz (0.56)	cc (0.98)
44	mile (0.2)	oz (0.24)	lb ⋅ ft (0.56)	m3/d (0.98)
45	kilograms (0.19)	m3 (0.24)	US gal (0.53)	miles per hour (0.97)
46	m3 (0.18)	yd (0.22)	psi (0.53)	bhp (0.92)
47	short tons (0.17)	short tons (0.22)	meters (0.52)	US gal (0.9)
48	square kilometers (0.16)	miles per hour (0.21)	cc (0.51)	imp gal (0.82)
49	yd (0.16)	nmi (0.18)	bhp (0.49)	lbf (0.81)
50	nmi (0.16)	square metres (0.15)	imp gal (0.48)	l (0.8)

The percentage of all units in the respective dataset is given in parentheses next to the surface form of the unit. As to be expected, Wiki-Quantities (small) and (tiny) are more balanced with respect to units. Unit counts are based on rule-based parsing of the last quantity in a quantity span.

Like Wiki-Quantities, we publish **Wiki-Measurements** in several variants, as well as in a raw format and pre-processed for SQuAD-style generative question-answering. Wiki-Measurements (large) contains 38 738 examples; the small variant contains 21 854 examples. The number of annotations per concept type, as well as the number of examples and pages from which they originate, is given in Table 4. Each example has an entity, a property, a value, and, if applicable, a unit annotation. Annotations for quantity modifiers and qualifiers are considered optional. Thus, quantitative statements may have additional qualifiers that are not annotated. 214 examples were manually curated to ensure complete annotations of the spatio-temporal scope and other qualifiers. The most frequent entities, properties, and units in the deduplicated (large) and balanced (small) variant of Wiki-Measurement are given in Tables 5 and 6, respectively.

**Table 3:** Comparing the English and Simple English Wikipedia as sources for Wiki-Quantities and Wiki-Measurements.

Dataset	Wikipedia Language	#Quantities	#Examples	#Wikipedia Pages
Wiki-Quantities (raw)	English	1 214 927	822 700	428 941
	Simple English	12 699	9 960	5 919
Wiki-Measurements (raw)	English	37 790	37 790	31 131
	Simple English	1 659	1 659	1 327

## Technical Validation

The quality of the datasets^4^ was primarily ensured by the approaches used to generate them and their elaborate, iteratively defined filter rules.

### Wiki-Quantites

Wiki-Quantities is created based on {{convert}} template calls. The {{convert}} templates are used to render quantities. Therefore, it is generally correct to consider their output as quantity span annotations. To be sure that all quantities in a sentence have been annotated, filter rules apply if there are numbers outside the template calls. Randomly sampling 100 examples of Wiki-Quantities (large) and manually validating them gave **100% precision and recall**. Nevertheless, the dataset contains incorrect examples. Therefore, we train a quantity span identification model on Wiki-Quantities (small) and other datasets, and compare the model outputs with the annotations for Wiki-Quantities (small), adjusting the annotations where necessary. We transfer the changes to Wiki-Quantities (tiny) and (large). As a superset, this leaves Wiki-Quantities (large) with more undetected mistakes than the smaller variants.

### Wiki-Measurements

Wiki-Measurements is created based on distant supervision which naturally yields noisy labels. However, we minimize noisy labels by only matching facts from the knowledge base (Wikidata) with texts related to the subject of the fact (its Wikipedia article). Furthermore, aligning the predicate (measured property) in addition to the subject and object further increases accuracy, but at the cost of neglecting expressions where the predicate is implicit. Therefore, implicit properties are considered if the value, unit, and entity matches provide sufficient evidence for a correct alignment. In addition, many more filtering rules ensure the correct alignment of facts with texts. However, distant supervision with quantitative facts is prone to several errors which we also observe:

- Often a sentence contains all the elements of a fact, but it is about a different thing that happens to share some of those elements.
- Numeric values occur more frequently and in more contexts than traditional entities in relation extraction, such as the name of a particular company or person^50^.
- Distant supervision with quantitative facts requires approximate matching due to different levels of rounding^50– 52^, which introduces false positives.
- Quantitative facts can be easily re-scoped (e.g., “literacy rate of *rural* India”^50^). Therefore, we extend matching entity and property spans to include modifiers such as ‘rural’, ‘minimum’, etc. In some cases, an entity or property is extended incorrectly.
- Time series in Wikidata (e.g., the growing population of a city) have many potential matches, making false positives more likely.

Randomly sampling 100 examples of Wiki-Measurements (large) and manually validating quadruples of  < entity, property, value, and unit > , yielded **84% accuracy when judged strictly and 94% accuracy** when essentially correct but not perfect examples were considered correct. Of these ten examples considered essentially correct, four are missing “number of” in a property span for a count (e.g., in “*In 2019, [Reipertswiller]*_*Entity*_*had [850]*_*Value*_ [*inhabitants*]_*Unit*_”, the property should be ‘number of inhabitants’, not ‘inhabitants’), three have an entity span that is too short (e.g., “[*Dalesbred*]_*Entity*_*ewes weigh*…”), two do not include an alternative name in the entity span (e.g., “[*Disentis*]_*Entity*_/*Mustér has an area*…”, and one is imprecise by describing a weightlifter’s weight class as actual mass.

Of the six incorrect examples, three contain the entity, property, and quantity of the Wikidata fact, but actually describe a different entity or property. For example, given the fact  < *Whittier, elevation above sea level*, 13 ± *1 metre*  > , the sentence is incorrectly annotated as “*The tsunami that hit [Whittier]*_*Entity*_*reached a [height]*_*Property*_*of 13 m ([43]*_*Value*_*[ft]*_*Unit*_) *and killed 13 people*.” Coincidentally, the height of the tsunami is about the same as the city’s elevation above sea level, colloquially height. In another example, a sculpture and the person it represents are confused, because they have the same name. Given the fact  < *William Penn, height, 37 foot*  > the sentence is incorrectly annotated as “*Due to* [*William Penn*]_*Entity*_*being a slave owner, columnist Stu Bykofsky for* “*The Philadelphia Inquirer*” *sardonically wrote*: “*We cant´ abide* [37]_*Value*_ [*feet*]_*Unit*_*of him towering over the city*. (*implicit property: height*)”. In one example, the property span is incorrectly extended ("[*Rolleston*]_*Entity*_*is described by Statistics New Zealand as a* [*medium urban area*]_*Property*_, *and covers* [8.53]_*Value*_ [*sq mi*]_*Unit*_”.), and one example is incorrect for multiple reasons ("90.60% *of the employed household members* ([17,257]_*Value*_[*work*]_*Unit*_*within the municipality of* [*Libon*]_*Entity*_. (*implicit property: number of households*)”).

### Limitations

Wiki-Quantities has several limitations. Although the {{convert}} template allows for diverse expressions of quantities, the variety is limited. In particular, the dataset does not include annotations of quantities with percentages as the unit of measurement (Wiki-Measurements does). Template calls that refer to Wikidata are ignored. Finally, common units such as kilogram, meter, etc. are overrepresented (see Table 2).

**Table 4:** Dataset statistics for different variants of Wiki-Measurements.

		Wiki-Measurements			
		large	large-strict	small	small-strict
Examples	#Pages	32 144	22 332	19 401	12 292
	#Total	38 738	26 009	21 854	13 257
	#Curated	237	197	237	197
	#Spatio-temporal Scopes Curated	217	190	217	190
	#Qualifiers Curated	214	188	214	188
Main Annotations	#Entities	38 758	26 012	21 873	13 260
	#Properties	30 346	25 978	15 050	13 226
	#Implicit Properties	17 675	8 173	12 498	5 361
	#Values	38 738	26 009	21 854	13 257
	#Units	36 301	23 917	20 429	12 018
Optional Annotations	#Quantity Modifiers	2 941	1 819	2 216	1 345
	#Temporal Scopes	1 875	1 624	728	557
	#Determination Methods	560	472	208	173
	#References	143	131	50	40
	#Locations	100	86	98	85
	#Applies to Part	65	34	56	30
	#Scopes	73	47	62	39
	#Start Times	24	8	24	8
	#Criteria Used	15	9	9	4
	#End Times	5	3	5	3
	#Other Qualifiers	394	138	259	106

Variants with added context are not included because they have the same statistics as their base variant.

The latter limitation also applies to Wiki-Measurements where certain entity classes, and hence certain properties and units, are over-represented (see Tables 5 and 6). Additionally, the use of bots in the creation of Wikipedia articles may lead to similar sentences about the same entity class (e.g., different cities, rivers, or mountains). These are removed in Wiki-Quantities, but retained in Wiki-Measurements. Also, unlike Wiki-Quantities, Wiki-Measurements does not support compound quantities (e.g., 2 ft 1 in). Finally, qualifiers and quantity modifiers are considered optional and are only sporadically annotated. Unlike many other datasets, Wiki-Quantities does not include quantity modifiers in the quantity span annotations.

**Table 5:** Top 50 most frequent entities, properties, values, units, and temporal scopes in Wiki-Measurements (large).

	Entities	Properties	Value	Units	Temporal Scope
1	it (2.83)	length (17.42)	three (0.79)	km2 (12.24)	2010 (46.24)
2	its (0.42)	area (17.42)	10 (0.66)	square miles (6.96)	2011 (12.0)
3	coin (0.11)	total area (11.95)	20 (0.62)	km (6.94)	2020 (8.48)
4	washington town…(0.07)	number of episodes (3.88)	two (0.6)	m (5.52)	2016 (3.47)
5	union township (0.07)	population (3.67)	13 (0.57)	mile (4.83)	2018 (3.47)
6	sata (0.06)	number of stories (2.93)	12 (0.52)	sq mi (4.22)	2017 (2.4)
7	lincoln township (0.06)	elevation (2.73)	30 (0.46)	metres (3.68)	2019 (2.19)
8	liberty township (0.06)	elevation above mean…(2.4)	four (0.44)	feet (2.89)	2014 (1.65)
9	coins (0.05)	number of seats (2.03)	40 (0.44)	episodes (2.64)	2015 (1.39)
10	jackson township (0.05)	diameter (1.44)	one (0.43)	mi (2.6)	2012 (0.85)
11	grant township (0.05)	total length (1.4)	26 (0.43)	$ (2.53)	2021 (0.8)
12	note (0.05)	height (1.19)	50 (0.42)	ft (2.35)	2001 (0.8)
13	richland township (0.04)	number of beds (1.02)	15 (0.41)	square kilom…(2.04)	2013 (0.69)
14	jefferson township (0.04)	average elevation (0.96)	100 (0.41)	kilometres (1.96)	2006 (0.64)
15	lent (0.04)	passengers (0.85)	5 (0.41)	square kilom…(1.94)	2007 (0.64)
16	town (0.04)	drainage basin (0.76)	60 (0.4)	story (1.82)	2000 (0.59)
17	center township (0.04)	land area (0.74)	2 (0.39)	miles (1.56)	2004 (0.43)
18	notes (0.04)	inhabitants (0.72)	16 (0.38)	seat (1.51)	1897 (0.32)
19	madison township (0.04)	capacity (0.71)	14 (0.36)	acts (1.1)	1999 (0.32)
20	three-dollar piece (0.04)	elevation above sea… (0.69)	32 (0.35)	kilometre (0.96)	2008 (0.27)
21	city (0.03)	absolute magnitude (0.68)	25 (0.34)	meters (0.95)	2005 (0.27)
22	water (0.03)	number of households (0.64)	3 (0.34)	acres (0.9)	2002 (0.21)
23	the amazon (0.03)	width (0.63)	1 (0.32)	kg (0.84)	1960 (0.21)
24	franklin township (0.03)	budget (0.62)	11 (0.32)	metre (0.83)	december 2019 (0.16)
25	cedar township (0.03)	altitude (0.61)	18 (0.31)	bed (0.8)	february 13, 1905 (0.16)
26	walnut township (0.03)	production budget (0.55)	8 (0.3)	mm (0.77)	july 24, 1936 (0.16)
27	kallichore (0.03)	total land area (0.53)	22 (0.3)	people (0.77)	2009 (0.16)
28	independence (0.03)	size (0.44)	24 (0.27)	years (0.76)	january 1, 2018 (0.16)
29	marion township (0.03)	distance (0.43)	6 (0.26)	hectares (0.74)	1992 (0.16)
30	gatwick (0.03)	surface area (0.42)	19 (0.25)	cm (0.67)	july 13, 1936 (0.16)
31	mercury (0.03)	atomic number (0.41)	5,000 (0.25)	households (0.66)	january 2020 (0.11)
32	three-dollar pieces (0.03)	number of employees (0.38)	4 (0.25)	kilometers (0.58)	1970 (0.11)
33	summit township (0.03)	prominence (0.38)	52 (0.25)	inhabitants (0.56)	1945 (0.11)
34	furlong (0.03)	albedo (0.34)	200 (0.25)	episode (0.56)	1984 (0.11)
35	fremont township (0.03)	box office (0.31)	23 (0.25)	days (0.53)	1814 (0.11)
36	triton (0.02)	episodes (0.31)	ten (0.23)	storey (0.53)	1989 (0.11)
37	garfield township (0.02)	weight (0.3)	17 (0.23)	minutes (0.53)	1 january 2018 (0.11)
38	2 bill (0.02)	seats (0.29)	75 (0.22)	passengers (0.53)	january 2018 (0.11)
39	the moon (0.02)	beam (0.28)	28 (0.22)	MW (0.48)	july 22, 1926 (0.11)
40	logan township (0.02)	total population (0.26)	500 (0.22)	minute (0.45)	december 31, 20…(0.11)
41	newfoundland (0.02)	orbital period (0.26)	five (0.22)	ha (0.39)	march 2014 (0.11)
42	she (0.02)	stories (0.25)	35 (0.22)	mg (0.39)	july 14, 1936 (0.11)
43	acre (0.02)	mass (0.25)	120 (0.22)	^∘^C (0.35)	august 2017 (0.11)
44	eden township (0.02)	beds (0.24)	36 (0.21)	tracks (0.35)	31 december 19…(0.11)
45	hurdles (0.02)	beats per minute (0.22)	21 (0.21)	^∘^ (0.32)	year 2021 (0.11)
46	germany (0.02)	inclination (0.21)	45 (0.21)	employees (0.31)	july 2020 (0.11)
47	finasteride (0.02)	altitude above sea l… (0.21)	300 (0.21)	acre (0.31)	june 29, 1994 (0.11)
48	long island (0.02)	seating capacity (0.2)	39 (0.2)	act (0.29)	1994 (0.11)
49	clay township (0.02)	basin area (0.15)	33 (0.2)	hours (0.28)	july 10, 1913 (0.11)
50	fairview township (0.02)	geographic area (0.14)	150 (0.2)	mg/m3 (0.26)	for the 2000 mil..(0.05)

For entities, properties and temporal scopes the case is ignored. The share in percent is given in parentheses.

**Table 6:** Top 50 most frequent entities, properties, values, units, and temporal scopes in Wiki-Measurements (small).

	Entities	Properties	Value	Units	Temporal Scope
1	it (1.43)	length (20.05)	three (1.08)	km (7.4)	2010 (16.48)
2	its (0.27)	area (9.55)	10 (0.91)	km2 (5.85)	2011 (11.95)
3	coin (0.18)	number of episodes (6.9)	20 (0.88)	m (5.66)	2020 (6.87)
4	coins (0.09)	number of stories (5.06)	two (0.86)	mile (4.38)	2017 (5.49)
5	note (0.08)	population (3.12)	13 (0.83)	episodes (4.09)	2018 (5.08)
6	lent (0.08)	number of seats (2.98)	12 (0.75)	metres (3.83)	2019 (4.4)
7	sata (0.07)	elevation (2.47)	four (0.69)	$ (3.02)	2016 (3.71)
8	notes (0.07)	total area (2.46)	one (0.67)	story (2.74)	2014 (3.43)
9	three-dollar piece (0.06)	number of beds (1.75)	40 (0.65)	ft (2.55)	2015 (2.88)
10	the amazon (0.05)	height (1.61)	30 (0.61)	sq mi (2.45)	2021 (1.79)
11	three-dollar pieces (0.05)	passengers (1.45)	26 (0.59)	mi (2.35)	2001 (1.65)
12	triton (0.04)	diameter (1.36)	50 (0.59)	kilometres (2.16)	2006 (1.51)
13	2 bill (0.04)	capacity (1.28)	60 (0.58)	miles (2.01)	2012 (1.37)
14	water (0.04)	inhabitants (1.0)	100 (0.56)	seat (1.9)	2000 (1.24)
15	the moon (0.04)	land area (0.95)	15 (0.56)	square kilom…(1.84)	2007 (1.24)
16	she (0.04)	budget (0.86)	16 (0.54)	feet (1.7)	2004 (0.96)
17	hurdles (0.04)	altitude (0.86)	2 (0.5)	kilometre (1.45)	2013 (0.69)
18	5 note (0.03)	total length (0.8)	14 (0.49)	square miles (1.41)	1999 (0.69)
19	gatwick (0.03)	distance (0.75)	25 (0.47)	acts (1.4)	1897 (0.55)
20	finasteride (0.03)	elevation above sea… (0.71)	5 (0.47)	bed (1.19)	december 2019 (0.41)
21	arcturus (0.03)	number of employees (0.68)	1 (0.46)	meters (1.18)	2008 (0.41)
22	mercury (0.03)	total land area (0.66)	11 (0.46)	mm (1.15)	2002 (0.41)
23	day (0.03)	size (0.63)	32 (0.44)	years (1.13)	2005 (0.41)
24	acre (0.03)	width (0.59)	18 (0.44)	metre (1.07)	1992 (0.41)
25	telescope (0.03)	episodes (0.56)	3 (0.43)	kg (1.05)	january 2020 (0.27)
26	aes (0.03)	seats (0.55)	22 (0.41)	cm (1.01)	1970 (0.27)
27	shvak (0.03)	box office (0.52)	8 (0.38)	hectares (0.93)	1945 (0.27)
28	10 bill (0.03)	weight (0.5)	24 (0.38)	acres (0.91)	1984 (0.27)
29	city (0.02)	drainage basin (0.5)	500 (0.36)	episode (0.91)	1989 (0.27)
30	fast (0.02)	stories (0.45)	52 (0.36)	minutes (0.88)	1 january 2018 (0.27)
31	frankfurt airport (0.02)	number of households (0.45)	19 (0.36)	storey (0.81)	july 24, 1936 (0.27)
32	6502 (0.02)	beds (0.43)	ten (0.35)	passengers (0.79)	january 2018 (0.27)
33	rack (0.02)	surface area (0.37)	five (0.33)	days (0.73)	2009 (0.27)
34	newfoundland (0.02)	mass (0.37)	200 (0.33)	minute (0.72)	december 31, 20…(0.27)
35	norethisterone (0.02)	prominence (0.37)	21 (0.32)	people (0.71)	march 2014 (0.27)
36	hanukkah (0.02)	absolute magnitude (0.35)	28 (0.32)	MW (0.7)	july 2020 (0.27)
37	pakistan (0.02)	seating capacity (0.35)	23 (0.32)	mg (0.68)	1960 (0.27)
38	howitzer m1 (0.02)	beam (0.32)	300 (0.31)	inhabitants (0.67)	1994 (0.27)
39	germany (0.02)	orbital period (0.29)	35 (0.31)	square kilom…(0.62)	july 13, 1936 (0.27)
40	sedna (0.02)	total population (0.28)	75 (0.3)	tracks (0.61)	july 10, 1913 (0.27)
41	beta pictoris (0.02)	altitude above sea l… (0.27)	39 (0.3)	ha (0.52)	for the 2000 mil..(0.14)
42	furlong (0.02)	apparent magnitude (0.25)	5,000 (0.29)	kilometers (0.51)	erected in 1979 (0.14)
43	raloxifene (0.02)	beats per minute (0.25)	17 (0.29)	employees (0.49)	current (0.14)
44	everest (0.02)	cost (0.24)	45 (0.29)	acre (0.49)	in january 2012…(0.14)
45	chain (0.02)	staff (0.24)	120 (0.27)	^∘^C (0.49)	now (0.14)
46	highway 1 (0.02)	half-life (0.23)	70 (0.27)	hours (0.45)	around 2005 (0.14)
47	titanic (0.02)	average elevation (0.21)	6 (0.27)	households (0.41)	on october 29,…(0.14)
48	today (0.02)	running time (0.21)	4 (0.27)	act (0.41)	officially open…(0.14)
49	alabama (0.02)	injured (0.21)	twelve (0.26)	meter (0.35)	started in 2009…(0.14)
50	naltrexone (0.02)	life expectancy (0.21)	29 (0.26)	hour (0.34)	finished in late…(0.14)

For entities, properties and temporal scopes the case is ignored. The share in percent is given in parentheses.

For each property, the quantitative statements were queried separately. To avoid hitting the timeout of the public Wikidata endpoint, a limit of 250 000 results is set. Thus, not all quantitative statements of Wikidata are obtained. This can be circumvented by not querying the public endpoint, but hosting a local copy, or writing a parser for the database dump. However, only few queries run into the timeout, and not setting the limit would result in a more unbalanced dataset.

Finally, parsing MediaWiki markup is imperfect. For example, many templates other than the {{convert}} template are not expanded, which may have introduced artifacts such as missing words or empty parentheses.

## Usage Notes

The easiest way to use the datasets^4^ unchanged for sequence labeling and question answering is to use the pre-processed versions for these tasks. If the use case is different or changes are desired, the raw datasets can be used as a starting point. To reproduce this work, the published code along with the accompanying Snakemake^38^ workflows can be used. Although the datasets are published in English, the methods are easily transferable to other languages. For those with more time and leisure, it is worth noting that the approach used to create Wiki-Quantities can be applied to scientific texts by using siunitx^53^ commands in LaTeX source files (e.g., from arXiv^54^) as annotations for quantities, numeric values, and units. When using or reproducing this work, please cite this data descriptor.

## Data Availability

The code for creating the datasets is available under open licenses at https://github.com/FZJ-IEK3-VSA/wiki-measurements.

## References

[1] Court, C. J. & Cole, J. M. Auto-generated materials database of Curie and Néel temperatures via semi-supervised relationship extraction. *Scientific Data* **5**, 180111 (2018). 29917013 10.1038/sdata.2018.111 PMC6007086

[2] Foppiano, L. *et al*. SuperMat: construction of a linked annotated dataset from superconductors-related publications. *Science and Technology of Advanced Materials: Methods* **1**, 34–44, 10.1080/27660400.2021.1918396 (2021).

[3] Göpfert, J., Kuckertz, P., Weinand, J., Kotzur, L. & Stolten, D. Measurement Extraction with Natural Language Processing: A Review. In *Findings of the Association for Computational Linguistics: EMNLP 2022*, 2191–2215 (Association for Computational Linguistics, Abu Dhabi, United Arab Emirates, 2022).

[4] Göpfert, J., Kuckertz, P., Weinand, J. M. & Stolten, D. Wiki-Quantities and Wiki-Measurements: Datasets of Quantities and their Measurement Context from Wikipedia 10.5281/zenodo.15462002 (2025).10.1038/s41597-025-05499-340695850

[5] Vrandečić, D. & Krötzsch, M. Wikidata: a free collaborative knowledgebase. *Communications of the ACM* **57**, 78–85, 10.1145/2629489 (2014).

[6] Wikidata. Wikidata Statistics https://www.wikidata.org/wiki/Wikidata:Statistics (2025).

[7] Wikidata. Airbus A380 https://www.wikidata.org/wiki/Q5830 (2024).

[8] Chinchor, N. A. Overview of MUC-7. In *Seventh Message Understanding Conference (MUC-7): Proceedings of a Conference Held in Fairfax, Virginia, April 29 - May 1, 1998* (1998).

[9] Grishman, R. & Sundheim, B. Design of the MUC-6 Evaluation. In *Sixth Message Understanding Conference (MUC-6): Proceedings of a Conference Held in Columbia, Maryland, November 6-8, 1995* (1995).

[10] Weischedel, R. *et al*. OntoNotes Release 5.0 10.35111/xmhb-2b84 (2013).

[11] Chen, C.-C., Huang, H.-H., Takamura, H. & Chen, H.-H. Numeracy-600K: Learning Numeracy for Detecting Exaggerated Information in Market Comments. In *Proceedings of the 57th Annual Meeting of the Association for Computational Linguistics*, 6307–6313, 10.18653/v1/P19-1635. (Association for Computational Linguistics, Florence, Italy, 2019).

[12] Lin, B. Y., Lee, S., Khanna, R. & Ren, X. Birds have four legs?! NumerSense: Probing Numerical Commonsense Knowledge of Pre-Trained Language Models. In *Proceedings of the 2020 Conference on Empirical Methods in Natural Language Processing (EMNLP)*, 6862–6868, 10.18653/v1/2020.emnlp-main.557 (Association for Computational Linguistics, Online, 2020).

[13] Spithourakis, G. P. & Riedel, S. Numeracy for Language Models: Evaluating and Improving their Ability to Predict Numbers. *Proceedings of the 56th Annual Meeting of the Association for Computational Linguistics (Volume 1: Long Papers)* 2104–2115, 10.18653/v1/P18-1196 (2018).

[14] Berg-Kirkpatrick, T. & Spokoyny, D. An Empirical Investigation of Contextualized Number Prediction. In *Proceedings of the 2020 Conference on Empirical Methods in Natural Language Processing (EMNLP)*, 4754–4764 (Association for Computational Linguistics, Online, 2020).

[15] Foppiano, L., Romary, L., Ishii, M. & Tanifuji, M. Automatic Identification and Normalisation of Physical Measurements in Scientific Literature. In *Proceedings of the ACM Symposium on Document Engineering 2019*, 1–4, 10.1145/3342558.3345411 (ACM, Berlin Germany, 2019).

[16] grobid-quantities (2015–2024). https://github.com/kermitt2/grobid-quantities. Swh:1:dir:dbf9ee55889563779a09b16f9c451165ba62b6d7.

[17] Harper, C. *et al*. SemEval-2021 Task 8: MeasEval - Extracting Counts and Measurements and their Related Contexts. In *Proceedings of the 15th International Workshop on Semantic Evaluation (SemEval-2021)*, 306–316, 10.18653/v1/2021.semeval-1.38 (Association for Computational Linguistics, Online, 2021).

[18] Almasian, S., Kazakova, V., Göldner, P. & Gertz, M. CQE: A Comprehensive Quantity Extractor. In Bouamor, H., Pino, J. & Bali, K. (eds.) *Proceedings of the 2023 Conference on Empirical Methods in Natural Language Processing*, 12845–12859, 10.18653/v1/2023.emnlp-main.793 (Association for Computational Linguistics, Singapore, 2023).

[19] Chaganty, A. & Liang, P. How Much is 131 Million Dollars? Putting Numbers in Perspective with Compositional Descriptions. In *Proceedings of the 54th Annual Meeting of the Association for Computational Linguistics (Volume 1: Long Papers)*, 578–587, 10.18653/v1/P16-1055 (Association for Computational Linguistics, Berlin, Germany, 2016).

[20] Thawani, A., Pujara, J. & Ilievski, F. Numeracy enhances the Literacy of Language Models. In *Proceedings of the 2021 Conference on Empirical Methods in Natural Language Processing*, 6960–6967 (Association for Computational Linguistics, Online and Punta Cana, Dominican Republic, 2021).

[21] Julavanich, T. & Aizawa, A. NumER: A Fine-Grained Numeral Entity Recognition Dataset. In Métais, E., Meziane, F., Horacek, H. & Kapetanios, E. (eds.) *Natural Language Processing and Information Systems*, Lecture Notes in Computer Science, 64–75, 10.1007/978-3-030-80599-97 (Springer International Publishing, Cham, 2021).

[22] Chen, C.-C., Huang, H.-H., Shiue, Y.-T. & Chen, H.-H. Numeral Understanding in Financial Tweets for Fine-grained Crowd-based Forecasting. *2018 IEEE/WIC/ACM International Conference on Web Intelligence (WI)* 136–143, 10.1109/WI.2018.00-97. (2018).

[23] Loukas, L. *et al*. FiNER: Financial Numeric Entity Recognition for XBRL Tagging. In *Proceedings of the 60th Annual Meeting of the Association for Computational Linguistics (Volume 1: Long Papers)*, 4419–4431, 10.18653/v1/2022.acl-long.303 (Association for Computational Linguistics, Dublin, Ireland, 2022).

[24] Chen, C.-C., Huang, H.-H. & Chen, H.-H. NumClaim: Investor’s Fine-grained Claim Detection. In *Proceedings of the 29th ACM International Conference on Information & Knowledge Management*, 1973–1976, 10.1145/3340531.3412100 (ACM, Virtual Event Ireland, 2020).

[25] Chen, C.-C., Huang, H.-H., Huang, Y.-L. & Chen, H.-H. Distilling Numeral Information for Volatility Forecasting. In *Proceedings of the 30th ACM International Conference on Information & Knowledge Management*, 2920–2924, 10.1145/3459637.3482089 (Association for Computing Machinery, New York, NY, USA, 2021).

[26] Foppiano, L., Dieb, T. M., Suzuki, A. & Ishii, M. Proposal for Automatic Extraction Framework of Superconductors Related Information from Scientific Literature. Tech. Rep., IEICE (2019).

[27] Friedrich, A. *et al*. The SOFC-Exp Corpus and Neural Approaches to Information Extraction in the Materials Science Domain. In *Proceedings of the 58th Annual Meeting of the Association for Computational Linguistics*, 1255–1268, 10.18653/v1/2020.acl-main.116 (Association for Computational Linguistics, Online, 2020).

[28] Mysore, S. *et al*. The Materials Science Procedural Text Corpus: Annotating Materials Synthesis Procedures with Shallow Semantic Structures. In *Proceedings of the 13th Linguistic Annotation Workshop*, 56–64, 10.18653/v1/W19-4007 (Association for Computational Linguistics, Florence, Italy, 2019).

[29] Wu, Y., Ting, H.-F., Lam, T.-W. & Luo, R. BioNumQA-BERT: answering biomedical questions using numerical facts with a deep language representation model. In *Proceedings of the 12th ACM Conference on Bioinformatics, Computational Biology, and Health Informatics*, BCB ’21, 1–6, 10.1145/3459930.3469557 (Association for Computing Machinery, New York, NY, USA, 2021).

[30] Chen, C.-C., Huang, H.-H. & Chen, H.-H. NQuAD: 70,000+ Questions for Machine Comprehension of the Numerals in Text. In *Proceedings of the 30th ACM International Conference on Information & Knowledge Management*, 2925–2929, 10.1145/3459637.3482155 (Association for Computing Machinery, New York, NY, USA, 2021).

[31] Chen, Z. *et al*. FinQA: A Dataset of Numerical Reasoning over Financial Data. In *Proceedings of the 2021 Conference on Empirical Methods in Natural Language Processing*, 3697–3711, 10.18653/v1/2021.emnlp-main.300 (Association for Computational Linguistics, Online and Punta Cana, Dominican Republic, 2021).

[32] Dua, D. *et al*. DROP: A Reading Comprehension Benchmark Requiring Discrete Reasoning Over Paragraphs. In *Proceedings of the 2019 Conference of the North American Chapter of the Association for Computational Linguistics: Human Language Technologies, Volume 1 (Long and Short Papers)*, 2368–2378, 10.18653/v1/2022.acl-long.303 (Association for Computational Linguistics, Minneapolis, Minnesota, 2019).

[33] Lathiff, N., Khloponin, P. P. & Bergler, S. CLaC-np at SemEval-2021 Task 8: Dependency DGCNN. In *Proceedings of the 15th International Workshop on Semantic Evaluation (SemEval-2021)*, 404–409, 10.18653/v1/2021.semeval-1.48 (Association for Computational Linguistics, Online, 2021).

[34] Subercaze, J. Chaudron: Extending DBpedia with Measurement. In Blomqvist, E.*et al*. (eds.) *The Semantic Web*, Lecture Notes in Computer Science, 434–448, 10.1007/978-3-319-58068-527 (Springer International Publishing, Cham, 2017).

[35] Hoffmann, R., Zhang, C. & Weld, D. S. Learning 5000 Relational Extractors. In *Proceedings of the 48th Annual Meeting of the Association for Computational Linguistics*, 286–295 (Association for Computational Linguistics, Uppsala, Sweden, 2010).

[36] Chia, Y. K., Bing, L., Aljunied, S. M., Si, L. & Poria, S. A Dataset for Hyper-Relational Extraction and a Cube-Filling Approach. In Goldberg, Y., Kozareva, Z. & Zhang, Y. (eds.) *Proceedings of the 2022 Conference on Empirical Methods in Natural Language Processing*, 10114–10133, 10.18653/v1/2022.emnlp-main.688 (Association for Computational Linguistics, Abu Dhabi, United Arab Emirates, 2022).

[37] Chen, J. *et al*. Timeline-based Sentence Decomposition with In Context Learning for Temporal Fact Extraction. In Ku, L.-W., Martins, A. & Srikumar, V. (eds.) *Proceedings of the 62nd Annual Meeting of the Association for Computational Linguistics (Volume 1: Long Papers)*, 3415–3432, 10.18653/v1/2024.acl-long.187 (Association for Computational Linguistics, Bangkok, Thailand, 2024).

[38] Mölder, F. *et al*. Sustainable data analysis with Snakemake. Tech. Rep. 10:33, F1000Research https://f1000research.com/articles/10-33 (2021). 10.12688/f1000research.29032.1 PMC8114187 34035898

[39] Wikipedia. Barn swallow (edit source) https://en.wikipedia.org/w/index.php?title=Barn_swallow&action=edit&oldid=1241593201 (2024).

[40] Wikipedia. Barn swallow https://en.wikipedia.org/w/index.php?title=Barn_swallow&oldid=1241593201 (2024).

[41] Wikipedia. Pyramid of Djoser (edit source) https://en.wikipedia.org/w/index.php?title=Pyramid_of_Djoser&action=edit&oldid=1242356270 (2024).

[42] Wikipedia. Pyramid of Djoser https://en.wikipedia.org/w/index.php?title=Pyramid_of_Djoser&oldid=1242356270 (2024).

[43] Montani, I. *et al*. explosion/spaCy: New Span Ruler component, JSON (de)serialization of Doc, span analyzer and more 10.5281/zenodo.6621076 (2022).

[44] Lo, K., Wang, L. L., Neumann, M., Kinney, R. & Weld, D. S2ORC: The Semantic Scholar Open Research Corpus. In Jurafsky, D., Chai, J., Schluter, N. & Tetreault, J. (eds.) *Proceedings of the 58th Annual Meeting of the Association for Computational Linguistics*, 4969–4983, 10.18653/v1/2020.acl-main.447 (Association for Computational Linguistics, Online, 2020).

[45] Wolf, T. *et al*. Transformers: State-of-the-Art Natural Language Processing. In Liu, Q. & Schlangen, D. (eds.) *Proceedings of the 2020 Conference on Empirical Methods in Natural Language Processing: System Demonstrations*, 38–45, 10.18653/v1/2020.emnlp-demos.6 (Association for Computational Linguistics, Online, 2020).

[46] Liu, Y. *et al*. RoBERTa: A Robustly Optimized BERT Pretraining Approach http://arxiv.org/abs/1907.11692 (2019).

[47] Levenshtein, V. I. Binary Codes Capable of Correcting Deletions, Insertions and Reversals. *Soviet Physics Doklady* **10**, 707 (1966).

[48] Mintz, M., Bills, S., Snow, R. & Jurafsky, D. Distant supervision for relation extraction without labeled data. In *Proceedings of the Joint Conference of the 47th Annual Meeting of the ACL and the 4th International Joint Conference on Natural Language Processing of the AFNLP*, 1003–1011, (Association for Computational Linguistics, Suntec, Singapore, 2009).

[49] Wikidata. Alabama https://www.wikidata.org/wiki/Q173 (2024).

[50] Madaan, A., Mittal, A., Mausam, Ramakrishnan, G. & Sarawagi, S. Numerical relation extraction with minimal supervision. In *Proceedings of the Thirtieth AAAI Conference on Artificial Intelligence*, AAAI’16, 2764–2771 (AAAI Press, Phoenix, Arizona, 2016).

[51] Vlachos, A. & Riedel, S. Identification and Verification of Simple Claims about Statistical Properties. In *Proceedings of the 2015 Conference on Empirical Methods in Natural Language Processing*, 2596–2601, 10.18653/v1/D15-1312. (Association for Computational Linguistics, Lisbon, Portugal, 2015).

[52] Intxaurrondo, A., Agirre, E., Lopez de Lacalle, O. & Surdeanu, M. Diamonds in the Rough: Event Extraction from Imperfect Microblog Data. In *Proceedings of the 2015 Conference of the North American Chapter of the Association for Computational Linguistics: Human Language Technologies*, 641–650, 10.3115/v1/N15-1066 (Association for Computational Linguistics, Denver, Colorado, 2015).

[53] Wright, J. siunitx - A comprehensive (SI) units package https://ctan.org/pkg/siunitx (2008–2025).

[54] Cornell University. arXiv.org https://arxiv.org/ (1991–2025).

[55] Wikipedia. Suldal https://en.wikipedia.org/w/index.php?title=Suldal&oldid=1267112000 (2025).

[56] Wikipedia. Triton (moon) https://simple.wikipedia.org/w/index.php?title=Triton_(moon)&oldid=9749756 (2024).

[57] Wikipedia. Colongra Power Station https://en.wikipedia.org/w/index.php?title=Colongra_Power_Station&oldid=1222955505 (2024).

